# Anal Canal Duplication in a 25-Year-Old Female Patient

**DOI:** 10.7759/cureus.36516

**Published:** 2023-03-22

**Authors:** Mauricio Gutierrez-Alvarez, Jorge Leal, Kevin Fuentes, Fernando Sotelo, Irving Fuentes, Daniel Camacho

**Affiliations:** 1 General Surgery, Medica Sur, Mexico City, MEX; 2 Surgery, Universidad Popular Autonoma del Estado de Puebla, Puebla, MEX; 3 Coloproctology, Medica Sur, Mexico City, MEX

**Keywords:** case report, congenital malformation, anal canal, perianal fistula, anal canal duplication

## Abstract

Anal canal duplication (ACD) is a congenital malformation that typically presents and is diagnosed early in life. It can be associated with other syndromes or congenital malformations. ACD is one of the rarest duplications of the gastrointestinal tract, with no more than 90 to 100 cases reported in the literature. It can be confused with more frequent pathologies such as perianal fistula, especially when it occurs in adulthood. We present the case of a 25-year-old female patient who presents with a second orifice above the native anal orifice. An arthroscopic examination was performed, an incidental diagnosis of ACD was made, and a complete excision of the duplicated anal canal was performed. The aim of the study is to expand the information on this rare pathology in order to take it into account as a differential diagnosis in patients with abscesses, recurrent fistulous tracts, or any other anorectal pathology.

## Introduction

Anal canal duplication (ACD) is a congenital malformation located in the midline above the native anal orifice [1,2]. It was first described in 1956 by Dukes et al. [1]; subsequently, Ochiai et al. defined it as a lesion that should include "a squamous epithelium at the caudal end, a transitional epithelium at the cranial end, and smooth muscle cells in the wall of the duplicated anal canal" [3]. ACD presents and is usually diagnosed early in life [4,5]. Thirty-six percent of cases may be associated with other malformations [4] such as myelomeningocele, midline defects [6,7], or most commonly, urinary malformations in up to 45% of cases [8]. It is even part of some rare syndromes such as Currarino syndrome, which include a characteristic triad associating anal stenosis, sacrococcygeal malformation, and presacral mass [1]. ACD is one of the rarest duplications of the gastrointestinal tract [9]. To our knowledge, as Ailhaud et al., Trecartin et al., and Özbey et al. had reported, no more than 90 or 100 cases had been reported in the literature to date [5,6,10]. Despite being a rare pathology, ACD should be considered in the differential diagnosis of other anorectal pathologies. What is relevant in our case is its late presentation and, due to the scarce information in the literature, the clinical picture may go unnoticed, confusing it with more frequent pathologies such as perianal fistulas [4]. The purpose of this case report is to broaden the existing information on this uncommon disease. 

## Case presentation

**Figure 1:**
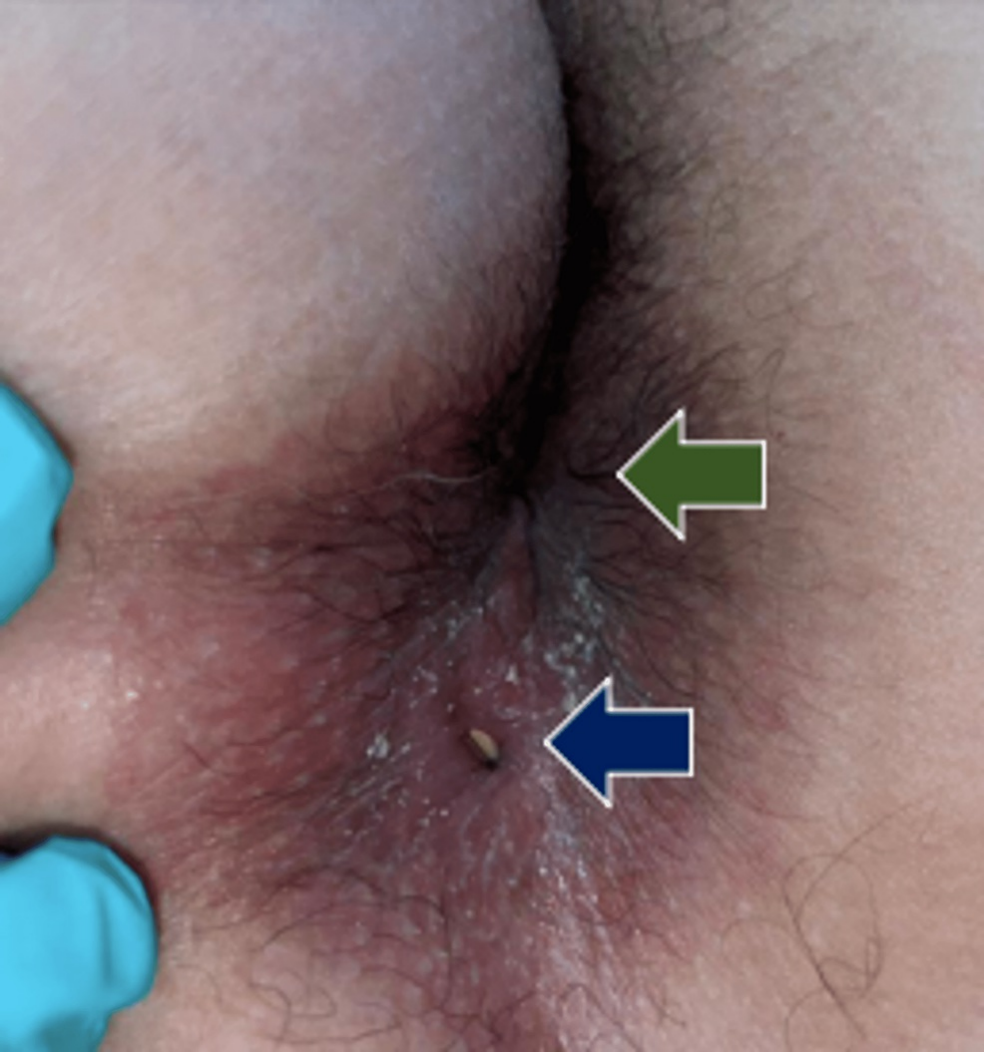
Secondary anal orifice (blue arrow) in the radius of 6 o'clock, 0.5 x 0.5 cm, from the native anal orifice (green arrow)

A 25-year-old female patient had spontaneous daily intermittent purulent fluid discharge through the perianal region for one month with no relieving or aggravating factors. Due to the persistent symptomatology, she presented as an outpatient for medical evaluation. Physical examination revealed a punctate lesion of 0.5 x 0.5 cm at approximately 3 cm in the 6 o'clock radius of the native anal orifice in the lithotomy position (Figure 1), with fecal matter inside and a foul odor. The rest of her examination, including the cardiovascular, respiratory, and neurological systems, showed no abnormalities. As part of her important medical history, she has been smoking for five years. She had an appendectomy five years before her admission. She denies any previous symptomatology, abscesses, or anorectal pathology. With regard to her birth history, her Apgar score was 9/10, no abnormalities were seen, and there were no other relevant details.

**Figure 2:**
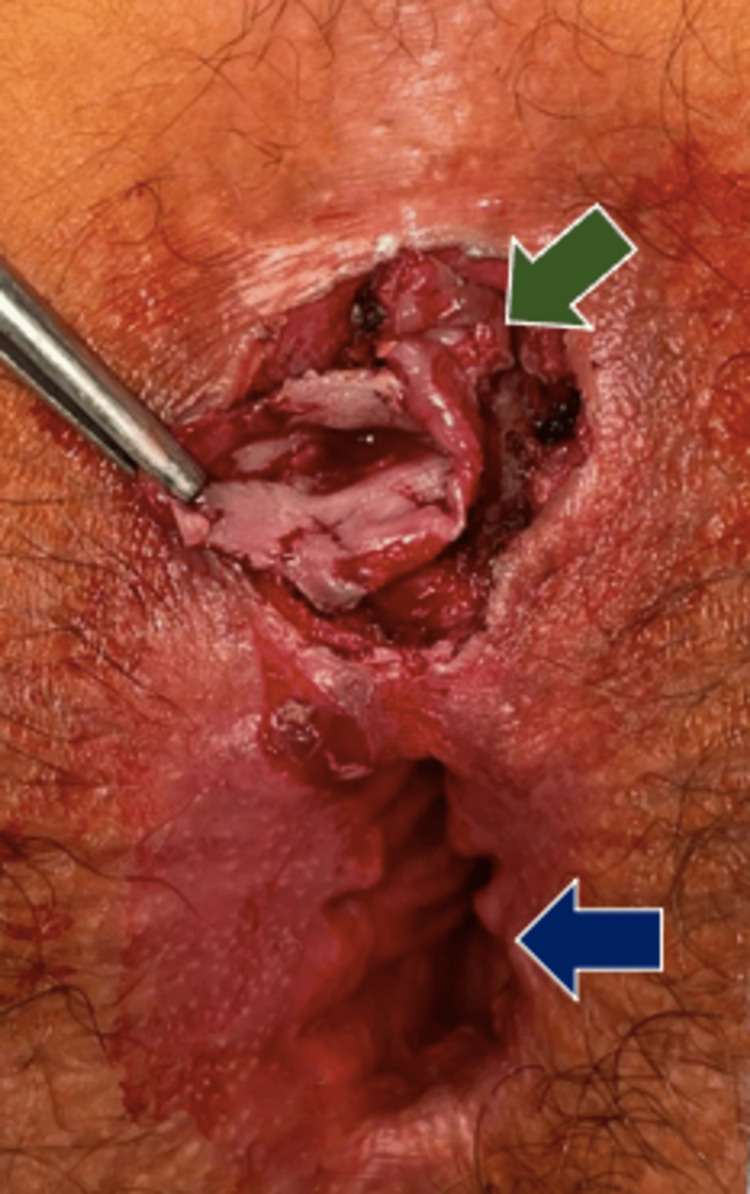
A complete resection of anal canal duplication (green arrow). Seen below is the native anal orifice (blue arrow).

**Figure 3:**
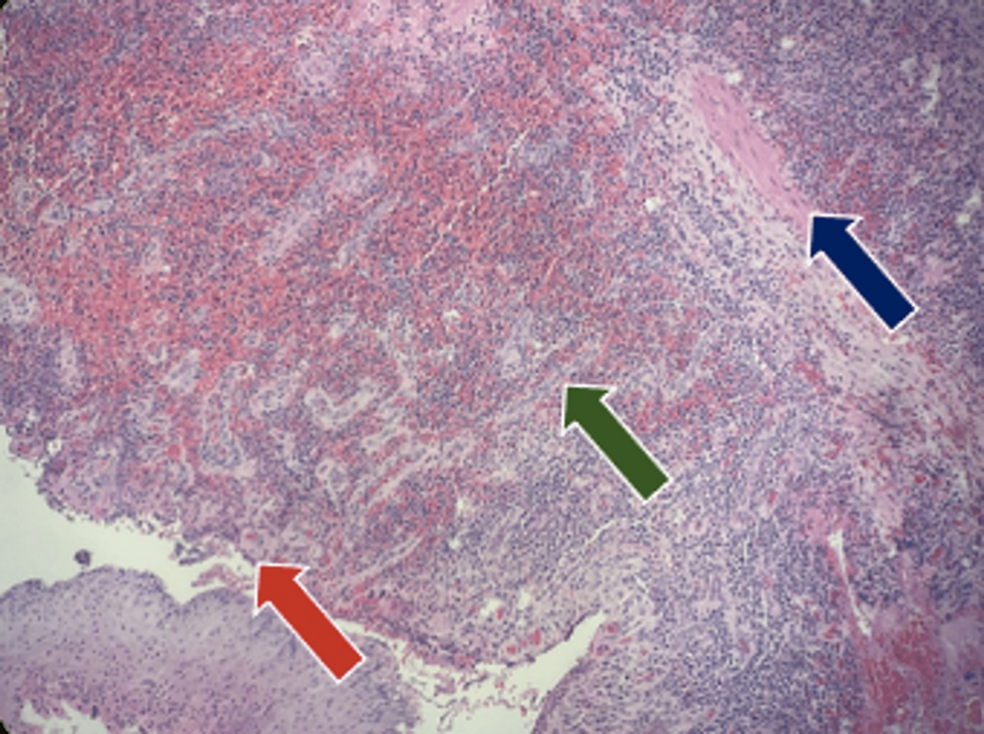
Histological examination of resected anal canal duplication with hematoxylin and eosin (H&E) staining showing smooth muscle fibers (blue arrow), transitional epithelium (green arrow), and stratified flat epithelium (red arrow).

With intermittent pain and purulent fluid discharge clinically mimicking a fistulous tract, the patient was scheduled for an anal exploration under general anesthesia. During the surgery, an anoscopy was performed and the anal canal was found to be intact, without lesions and without a fistulous tract between the orifice located at 6 o'clock in the lithotomy position and the native anal orifice. The secondary orifice was explored, finding smooth muscle fibers that contract with the application of energy (Video 1), suggestive of an anal canal duplication. Complete resection of the accessory anal canal was performed, followed by the closure of the incision from the deeper tissues to the surface and then an uncomplicated Z-plasty (Figure 2). The histopathology report found smooth muscle fibers and stratified flat epithelium with an apparent transitional epithelium between them (Figure 3). The patient was discharged 72 hours after the surgery. At the 12-month follow-up, the patient continued to make adequate progress with no recurrences.

**Video 1:** On the upper side of the image, smooth muscle fibers are in the secondary orifice, which contract in a sphincter-like manner with the thermal cautery. Under the secondary orifice, the native anal canal is seen.

## Discussion

ACD is a congenital malformation that typically manifests early in life [4,5], but in a minority of cases such as the current case, it manifests in adulthood [1]. In a compilation of case series and case reports with a total of 72 patients, the maximum age was 14 years [6], much younger than the case we present. The oldest age found in the literature is a 67-year-old male described by Toyonaga et al. [11].

ACD occurs more commonly in females, with a predilection of nine females for every male [1,4,6]. Clinically, it can be diagnosed early in life through a complete physical examination where an accessory orifice of approximately 2-5 mm in diameter is seen above the native anal orifice at the 6 o'clock radius [1,4,8,12], as is the lesion found in our patient.

ACD tends to be asymptomatic in the first two years of life, and thereafter, the incidence of clinical manifestations increases [5]. Clinical manifestations occur when there is some type of complication, such as infection, and they simulate recurrent abscesses or fistulas [4,5]; thus, a differential diagnosis will have to be made, especially in adult patients. Age is proportional to the probability of presenting clinical manifestations; in fact, in the largest adult case series reported, described by Mirzaei et al., which includes four patients (20, 24, 35, and 50 years of age), all of them had some clinical manifestation. Three of them presented with perianal abscesses and one of them with a recurrent fistula [2].

The most useful diagnostic studies are fistulography, magnetic resonance imaging, and anoscopy [4,7]; however, a surgical specimen with a histopathology report is always necessary [7,8]. In our case, the histopathology findings showed stratified epithelium and the presence of smooth muscle fibers, and inflammation associated with transitional epithelium was observed, as reported in other cases in the literature [3]. The histopathology findings, together with the clinical presentation observed where there is contraction of muscle fibers that resemble those of a sphincter, helped to integrate the diagnosis of ACD. Few cases have reported anal glands in surgical specimens; the most recent to our knowledge is that of Tudor et al. [4].

ACD has benign behavior, and only one case of malignant transformation has been reported in history [4]. However, the prevention of complications and clinical manifestations in the future justifies surgical treatment early in life [2,4,6,10]. There are two surgical therapeutic options: excision of the mucosa and surgical abolition of the remaining canal or complete excision of the ACD [2,4,5,12]. In our case, we opted for complete resection in order to avoid possible complications or recurrence in the future. In the medium-term follow-up of 12 months, a good result has been obtained without recurrence or complications.

## Conclusions

Duplication of the anal canal is a rare malformation with few cases described in the literature, mostly in pediatric patients. It is the physician's task to keep in mind this diagnostic possibility in patients with recurrent abscesses or fistulous tracts, especially in adulthood and in pediatric patients with a secondary orifice close to the native anal orifice. The therapeutic option should be surgery, which should be performed as soon as possible, even if the patients are asymptomatic, in order to avoid complications such as malignant transformation. Finally, it is necessary to intentionally look for and be alert to probable associated malformations.

## References

[1] Karamatzanis I, Kosmidou P, Harmanis S, Karamatzanis I, Harmanis G (2022). Early diagnosis of anal canal duplication: the importance of a physical examination. Cureus.

[2] Mirzaei R, Mahjubi B, Alvandipoor M, Karami MY (2015). Late presentation of anal canal duplication in adults: a series of four rare cases. Ann Coloproctol.

[3] Ochiai K, Umeda T, Murahashi O, Sugitoh T (2002). Anal-canal duplication in a 6-year-old child. Pediatr Surg Int.

[4] Mateescu T, Tarta C, Stanciu P, Dema A, Lazar F (2021). Anal canal duplication in an adult female-case report and pathology guiding. Medicina (Kaunas).

[5] Ailhaud A, Alao O, Sole Cruz E (2021). Anal canal duplication in children: a monocentric experience of 12 cases. Pediatr Surg Int.

[6] Trecartin AC, Peña A, Lovell M, Bruny J, Mueller C, Urquidi M, Bischoff A (2019). Anal duplication: is surgery indicated? A report of three cases and review of the literature. Pediatr Surg Int.

[7] Li D, Liu S, Feng J, Yang J (2022). Anal canal duplication mimicking recurrent abscess: a case report and review of the literature. Front Surg.

[8] Narci A, Dilek FH, Cetinkurşun S (2010). Anal canal duplication. Eur J Pediatr.

[9] Honda S, Minato M, Miyagi H, Okada H, Taketomi A (2017). Anal canal duplication presenting with abscess formation. Pediatr Int.

[10] Özbey H (2021). Anal canal duplication in a 12-year-old girl. J Pediatr Gastroenterol Nutr.

[11] Toyonaga T, Matsuda H, Mibu R, Tominaga Y, Hirata K, Takeyoshi M, Tsuneyoshi M (2018). Anal canal duplication associated with presacral cyst in an adult. J Anus Rectum Colon.

[12] Akova F, Altinay S, Aydin E (2020). The controversy of surgical intervention for anal canal duplication in children. Pak J Med Sci.

